# Temporal trends in the pre-procedural TIMI flow grade among patients with ST- segment elevation myocardial infarction – From the ACSIS registry

**DOI:** 10.1016/j.ijcha.2021.100868

**Published:** 2021-09-01

**Authors:** Nili Schamroth Pravda, Tal Cohen, Robert Klempfner, Ran Kornowski, Roy Beigel, Katia Orvin, Merry Abitbol, Miri Schamroth Pravda, Idit Dobrecky-Mery, Ronen Rubinshtein, Madji Saada, Alon Eisen

**Affiliations:** a Department of Cardiology, Rabin Medical Center, Petach Tikva, Israel; b Sackler Faculty of Medicine, Tel Aviv University, Tel Aviv, Israel; c Israeli Center of Cardiovascular Research, Tel Hashomer, Israel; d The Heart Center, Chaim Sheba Medical Center, Tel Hashomer, Israel; e Internal Medicine A, Meir Medical Center, Kfar Saba, Israel; f Bnai Zion Medical Center, Haifa, Israel; g Wolfson Medical Center, Holon, Israel; h Hilel Yaffe Medical Center, Hadera, Israel

**Keywords:** STEMI, TIMI flow grade, Temporal trends

## Abstract

**Background:**

Pre-procedural TIMI coronary flow grade in patients with ST segment elevation myocardial infarction (STEMI) is associated with adverse clinical outcomes. There have been great advances in pharmacologic and invasive treatment of STEMI patients in the current era. We aimed to assess the temporal trends in clinical outcomes according to the TIMI flow grade amongst these patients.

**Methods:**

Data of patients with STEMI from the acute coronary syndrome Israeli Survey (ACSIS) registry. A time-dependent analysis stratifying patient by TIMI flow grade 0 and TIMI flow grade 1–3 was performed. Survey years were divided to early (2008–2010) and late period (2013–2018). Clinical outcomes included in-hospital complications, 30d MACE (death, myocardial infarction, stroke, unstable angina, stent thrombosis, urgent revascularization) and 1-year mortality.

Results and Conclusions: Included were 2453 patients. The majority of patients had pre-procedural TIMI flow 0 (58.9% in the early period and 58.7% in the late period, P = 0.97). In-hospital complications of patients with TIMI flow 0 has significantly decreased over time (36.1% vs 26.8%, P < 0.001) but not amongst patients with TIMI flow 1–3. Compared with TIMI flow 1–3, patients with TIMI flow 0 had worse 30d MACE and 1-year mortality. There was no temporal change of these outcomes in either TIMI flow grade group. TIMI flow grade 0 is still more common among patients with STEMI and is associated with poorer prognosis. Nevertheless, over time, in-hospital complications have decreased among patients with TIMI 0, while 30d MACE and 1-year mortality has remained unchanged.

## Highlights

• Preprocedural TIMI flow grade remains of prognostic significance.
• Patients with TIMI 0 have a poorer prognosis than their counterparts with TIMI 1-3.
• In-hospital complications have decreased among patients with TIMI 0 over time.
• 30-d MACE and 1-year mortality remained unchanged in those with TIMI 0 or 1-3.

## 1 Introduction

Thrombolysis in Myocardial Infarction (TIMI) coronary grade flow is an established, validated score to assess the epicardial perfusion on coronary angiography. TIMI flow grade 0 represents total occlusion, while TIMI flow grade 3 represents normal epicardial perfusion [1]. Previous studies have demonstrated that patients with low grade TIMI flow prior to percutaneous coronary intervention (PCI) have a less favorable outcome. Preprocedural TIMI flow has been found to be an independent predictor of survival in patients with acute myocardial infarction [2], [3] and has also been shown to predict final infarct size [4]. Over the years there has been marked advancement in the management of patients presenting with STEMI in the areas of pre-hospital management, pharmacological therapy and procedural techniques. However, there is conflicting evidence if these advances have translated into improved clinical outcomes [5], [6]. We aimed to assess the temporal trends in preprocedural TIMI flow grade among patients presenting with STEMI and to examine whether TIMI flow grade is associated with clinical outcomes and remains a significant prognostic factor in the current era.

## 2 Methods

The current study included consecutive patients from the acute coronary syndrome (ACS) Israeli Surveys (ACSIS) between 2008 and 2018, who presented with STEMI and had data on the initial TIMI grade prior to revascularization. The ACSIS registry is a prospective survey conducted every 2–3 years that enrols consecutive patients from all 26 coronary care units operating in Israel over a 2-month period. The data is entered electronically by dedicated and specifically trained research personnel. Informed consent is obtained by all patients. The pre-specified demographic, cardio-vascular risk factors, co-morbidities, medications and clinical data were recorded along with admission and discharge diagnoses as defined by the attending physicians based on clinical, electrocardiographic, and biochemical criteria. The institutional review board (IRB) of all the participating hospitals approved the survey, which was performed in accordance with the Helsinki declaration. Preprocedural TIMI coronary grade flow was determined by the treating physician at the time of angiography (TIMI flow grade 0, 1, 2, or 3). Patients’ management was at the discretion of the attending physicians. A time-dependent analysis of patients with TIMI flow grade 0 versus TIMI flow grade 1–3 was performed. Survey years were divided according to the time periods of the index ACS event: early (2008–2010) and late period (2013–2018). Clinical outcomes included in-hospital complications, 30-day major adverse cardiovascular events (MACE), and 1-year all-cause mortality. In- hospital complications were a composite of congestive heart failure, hemodynamically significant right ventricle infarction (RVI), recurrent myocardial infarction (MI), stent thrombosis, ventricular septal defect, ≥ moderate mitral regurgitation, pericarditis, ventricular arrhythmia, new onset atrial fibrillation, bradycardia/asystole, cerebrovascular event and acute renal failure. 30-day MACE was comprised of all-cause mortality, MI, stroke, unstable angina, stent thrombosis and urgent revascularization. Data regarding the outcomes were determined by hospital chart review, telephone contact, clinical follow-up and by matching identification numbers of patients with the Israeli National Population Registry (for 30-day and 1-year mortality).

## 3 Statistical Methods

Patients characteristics are presented as mean (SD) or median (IQR) as appropriate for normal/non-normal distributed continuous variables, and as frequency (%) for categorical variables. The study groups were tested with chi-square for categorical variables and with *t*-test or Mann Whitney Wilcoxon test as appropriate for normal/nonnormal distributed continuous variables. The Kaplan-Meier log rank test was used to test the variable of interest on survival. All tests were conducted at a two sided overall 5% significance level (alpha = 0.05).

## 4 Results

**Fig. 1:**
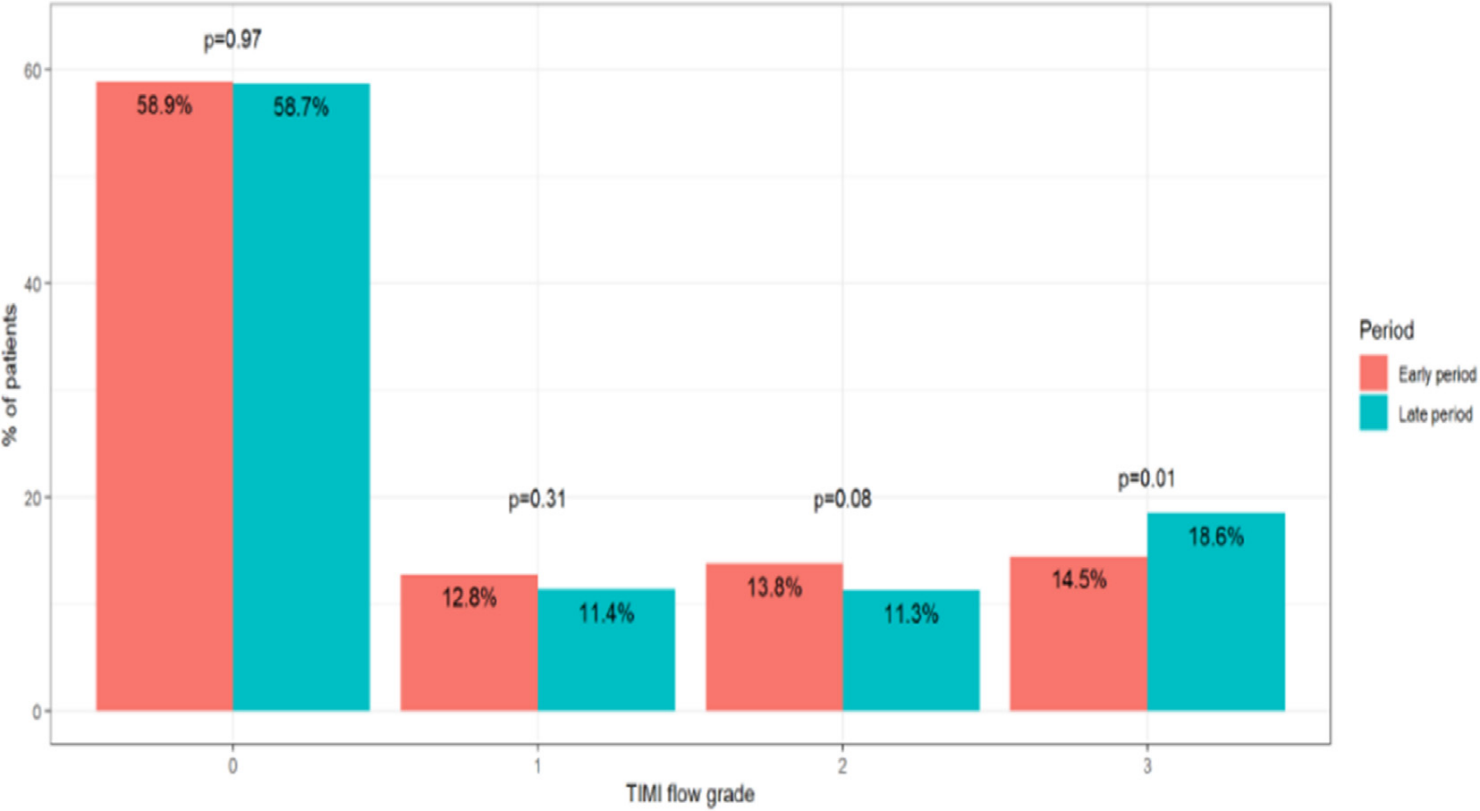
The distribution of preprocedural TIMI flow grades in the early (2008–2010) and late periods (2013–2018).

Of the 3840 patients presenting with STEMI and who underwent primary PCI, included were 2453 patients for whom data regarding TIMI flow prior to revascularization was available. There were 934 (38.1%) patients in the early period and 1519 (61.9%) patients in the late period. The distribution of preprocedural TIMI flow grades in the early and late periods is depicted in Fig. 1. The majority of patients presenting with STEMI had TIMI flow 0 (58.9% in the early period and 58.7% in the late period, P = 0.97). In the late period, there were more patients with STEMI presenting with TIMI flow 3 compared to the earlier period (18.6% vs 14.5%, P = 0.01). Baseline characteristics are depicted in Table 1 and Table S1. Demographic data was similar between those with TIMI flow 0 and TIMI flow 1–3. The median age of the patients was 60 years, and the majority were male (82.2% vs 83.6%, P = 0.39). Comorbidities, such as dyslipidemia were highly prevalent in both groups (67.7% vs 64.8%, P = 0.15), as well as increased BMI (27.1 vs 26.8, P = 0.12) and almost a quarter of the patients had a prior MI (24.0% vs 21.1%, P = 0.11). Prior use of medications such as angiotensin receptor blockers (ARBS) and beta blockers was more prevalent in those presenting with TIMI 0 than those with TIMI 1-3. Over time, among patients with TIMI 0, cardiovascular risk-factors were similar. Among those with TIMI 1-3, the prevalence of hypertension and diabetes increased significantly (46.3% vs. 54.3%, P = 0.12, and 33.2% vs 25.6%, P = 0.01, in the early and late periods, respectively). Over time there was also a significant increase in the use of ARBs, statins and hypoglycemic agents in both those presenting with TIMI 0 as well as in those presenting with TIMI 1-3.

**Table 1:** Baseline Characteristics over the time periods of those with TIMI 0 vs TIMI 1-3.

	TIMI 0			TIMI 1-3		
	2008–2010	2013–2018	p value	2008–2010	2013–2018	p value
n	550	892		384	627	

*Baseline characteristics*						
Age, years (median [IQR[)	59.00 [52.00, 69.00]	60.00 [53.00, 69.00]	0.40	58.00 [51.00, 68.00]	60.00 [52.00, 71.00]	0.06
Gender (male)	449 (81.6)	736 (82.5)	0.72	323 (84.1)	522 (83.3)	0.78
Dyslipidemia	375 (68.8)	594 (67.0)	0.52	257 (67.1)	394 (63.4)	0.26
Hypertension	290 (53.0)	486 (54.7)	0.57	177 (46.3)	338 (54.3)	0.01
Current smokers	268 (49.0)	463 (51.9)	0.30	197 (51.8)	295 (47.0)	0.15
Diabetes mellitus	161 (29.3)	278 (31.2)	0.48	98 (25.6)	207 (33.2)	0.01
Family history of CAD	167 (31.7)	261 (33.2)	0.61	123 (33.3)	188 (34.6)	0.74
BMI (kg/m2), (median [IQR([	26.88 [24.49, 29.63]	27.45 [24.69, 30.39]	0.20	26.67 [24.44, 29.41]	26.95 [24.38, 29.76]	0.88
Prior MI	132 (24.0)	213 (23.9)	1.00	82 (21.4)	131 (20.9)	0.90
Prior CABG	15 (2.7)	24 (2.7)	1.00	13 (3.4)	15 (2.4)	0.45
Prior PCI	134 (24.5)	213 (23.9)	0.86	86 (22.5)	134 (21.4)	0.72
Chronic renal failure	23 (4.2)	49 (5.5)	0.33	20 (5.2)	44 (7.0)	0.31
PVD	33 (6.0)	35 (3.9)	0.09	24 (6.3)	26 (4.2)	0.18
Prior CVA/TIA	26 (4.7)	59 (6.6)	0.17	29 (7.6)	46 (7.4)	1.00
History of CHF	24 (4.4)	31 (3.5)	0.46	7 (1.8)	17 (2.7)	0.49

*Prior medications*						
Aspirin	192 (35.2)	307 (36.6)	0.64	129 (33.9)	205 (35.0)	0.75
Clopidogrel	35 (6.4)	60 (7.4)	0.54	21 (5.5)	26 (4.8)	0.74
ACE-I	131 (24.1)	188 (23.8)	0.95	76 (19.9)	127 (23.6)	0.21
ARB	34 (6.2)	102 (14.1)	<0.001	19 (5.0)	47 (9.8)	0.01
Beta blockers	140 (25.7)	207 (26.2)	0.89	86 (22.6)	116 (21.3)	0.71
Statins	204 (37.5)	329 (46.9)	0.001	132 (34.6)	219 (46.3)	0.001
CCB	83 (15.3)	140 (19.2)	0.07	51 (13.4)	70 (14.6)	0.68
Nitrates	27 (5.0)	14 (2.0)	0.005	7 (1.8)	7 (1.5)	0.87
Hypoglycemic agents	84 (15.3)	172 (19.3)	0.06	52 (13.5)	128 (20.4)	0.007
Diuretics	66 (12.0)	64 (8.8)	0.07	31 (8.1)	40 (8.4)	0.96

CAD = coronary artery disease, IQR = interquartile range, BMI = body mass index, MI = myocardial infarction, CABG = coronary artery bypass graft surgery, PCI = percutaneous intervention, PVD = peripheral vascular disease, CVA = cerebral vascular accident, TIA = transient ischemic attack, CHF = congestive heart failure, ACE-I = Angiotensin-converting-enzyme inhibitor, ARB = Angiotensin II receptor blocker, CCB = calcium channel blockers.

Patients' vital signs on first medical contact are shown in Table 2 and Table S2. Time from symptom onset to primary PCI in patients with TIMI 0 was similar to patients with TIMI 1-3 (184.0 min vs 194.5 min, P = 0.13). This did not significantly change over time. Table 3 and Table S3 show the angiography, in-hospital complications and laboratory results. Patients presenting with TIMI 0 were significantly more likely to have an in-hospital complication compared with TIMI 1-3 (30.3% vs 21.5%, P < 0.001), including cardiogenic shock (6.3% vs 3.7%, P = 0.005), hemodynamically significant RVI (2.8% vs 0.7%, P < 0.001), as well as arrhythmic complications such as ventricular tachycardia (2.6% vs 0.6%, P < 0.001), new atrial fibrillation (7.1% vs 4.1%, P = 0.002), high degree atrioventricular block (4.1% vs 2.3%, P = 0.019) and asystole (3.1% vs 1.4%, P = 0.009). Over time, there was a significant increase in the use of drug eluting stents and a decrease in the use of bare metal stents. Over time, there was a significantly lower rate of in-hospital complications in those presenting with TIMI 0 (36.1% in the early period vs. 26.8% in the late period, P < 0.001), mainly driven by less patients presenting with Killip class 2–3, RVI, and some arrhythmias. This difference over time periods was not observed in those presenting with TIMI 1-3 in whom the rate of in-hospital complications remained unchanged. Patients with TIMI 0 had a significantly higher rate of acute renal failure and a higher biomarker rise compared to those presenting with TIMI 1-3. Table 4 and S4 details the treatment at discharge of the patients. There was no significant difference between the treatment prescribed to those presenting with TIMI 0 vs TIMI 1-3. Regardless of the initial TIMI flow, the majority of patients were discharged with clopidogrel in the early period and with newer P2Y12 inhibitor agents in the late period. Over time, significantly more patients were discharged with statins and ACE-I (Angiotensin-converting-enzyme inhibitors) /ARBs regardless of initial TIMI score, and more patients were referred for cardiac rehabilitation on discharge in the late period.

**Table 2:** Vital Signs on first medical contact over time of those with TIMI 0 vs TIMI 1-3.

	TIMI 0			TIMI 1-3		
	2008–2010	2013–2018	p value	2008–2010	2013–2018	p value
n	550	892		384	627	
Killip Class I	479 (87.1)	753 (87.9)	0.97	345 (89.8)	548 (90.3)	0.86
Killip Class II	37 (6.7)	53 (6.2)		18 (4.7)	24 (4.0)	
Killip Class III	13 (2.4)	20 (2.3)		12 (3.1)	17 (2.8)	
Killip Class IV	21 (3.8)	31 (3.6)		9 (2.3)	18 (3.0)	
Heart rate (bpm) (median [IQR])	74.00 [63.00, 88.00]	78.00 [67.00, 90.00]	0.002	76.00 [64.00, 90.00]	79.00 [67.00, 90.00]	0.15
Systolic Blood Pressure (mmHg) (median [IQR])	136.00 [116.00, 152.00]	138.50 [120.00, 157.00]	0.10	135.00 [118.00, 157.00]	140.00 [120.00, 159.00]	0.05
Diastolic Blood Pressure (mmHg) (median [IQR])	80.00 [70.00, 91.00]	80.00 [70.00, 93.00]	0.14	80.00 [70.00, 90.00]	80.50 [71.00, 94.00]	0.04
Sinus Rhythm	497 (90.4)	754 (84.5)	0.002	359 (93.5)	546 (87.1)	0.002
AF/SVT	19 (3.5)	49 (5.5)	0.10	9 (2.3)	30 (4.8)	0.07
VT/VF	5 (0.9)	20 (2.2)	0.09	6 (1.6)	12 (1.9)	0.86
2nd/3rd degree Atrioventricular Block	8 (2.5)	23 (2.6)	1.00	5 (2.5)	7 (1.1)	0.29
Time from symptoms onset to primary PCI (in STEMI patients) (median [IQR])	195.00 [128.00, 330.00]	180.00 [125.00, 302.50]	0.21	200.00 [135.75, 316.25]	190.00 [125.00, 330.00]	0.50

AF/SVT = Atrial fibrillation/Supraventricular Tachycardia, VT/VF = Ventricular Tachycardia/Ventricular Fibrillation, PCI = Percutaneous Coronary Intervention, STEMI = ST-elevation myocardial infarction.

**Table 3:** Angiography, In hospital complications and laboratory tests over time periods of those with TIMI 0 vs TIMI 1-3.

	TIMI 0			TIMI 1-3		
	2008–2010	2013–2018	p value	2008–2010	2013–2018	p value
n	550	892		384	627	

*Angiography*						
Non-obstructive	6 (1.1)	51 (5.9)	<0.001	4 (1.0)	43 (7.1)	<0.001
1 Vessel Diseased	214 (39.1)	375 (43.1)		145 (38.0)	246 (40.6)	
2 Vessel Diseased	188 (34.4)	264 (30.3)		136 (35.6)	178 (29.4)	
3 Vessels Diseased	139 (25.4)	180 (20.7)		97 (25.4)	139 (22.9)	
Left Anterior Descending Artery	266 (48.5)	401 (45.2)	0.34	177 (46.2)	300 (48.4)	0.13
Circumflex Artery	67 (12.2)	121 (13.6)		52 (13.6)	93 (15.0)	
Right Coronary Artery	204 (37.2)	347 (39.1)		141 (36.8)	216 (34.8)	
Left Main	4 (0.7)	4 (0.5)		4 (1.0)	5 (0.8)	
Saphenous Vein Graft	8 (1.5)	8 (0.9)		4 (1.0)	5 (0.8)	
Ramus	0 (0)	4 (0.5)		5 (1.3)	0 (0)	
Other Graft	0 (0)	2 (0.2)		0 (0)	1 (0.2)	
Bare Metal Stent	434 (87.1)	139 (16.7)	<0.001	297 (85.1)	121 (20.5)	<0.001
Drug Eluting Stent	86 (17.3)	651 (78.4)	<0.001	64 (18.3)	470 (79.5)	<0.001

*In-hospital complications*						
Composite of In-hospital complications	197 (36.1)	236 (26.8)	<0.001	89 (23.4)	127 (20.3)	0.28
CHF mild-moderate (Killip Class II)	58 (10.6)	55 (6.2)	0.004	24 (6.2)	31 (5.0)	0.46
Pulmonary edema (Killip III)	35 (6.4)	29 (3.3)	0.008	14 (3.6)	23 (3.7)	1.00
Cardiogenic shock (Killip IV)	39 (7.1)	52 (5.9)	0.41	16 (4.2)	21 (3.3)	0.61
Hemodynamically significant Right Ventricular Infarction	27 (4.9)	13 (1.5)	<0.001	6 (1.6)	1 (0.2)	0.02
Repeat MI	7 (1.3)	6 (0.7)	0.37	1 (0.3)	3 (0.5)	0.98
Stent thrombosis (definite/probable/possible)	13 (2.4)	14 (1.6)	0.38	6 (1.6)	6 (1.0)	0.57
VSD	1 (0.2)	2 (0.2)	1.00	1 (0.3)	0 (0.0)	0.80
MR moderate - severe	10 (1.8)	14 (1.6)	0.88	3 (0.8)	6 (1.0)	1.00
Pericarditis	4 (0.7)	12 (1.3)	0.40	1 (0.3)	3 (0.5)	0.98
Sustained VT (>125 bpm)	14 (2.5)	24 (2.7)	0.99	4 (1.0)	2 (0.3)	0.30
Primary VF	25 (4.5)	30 (3.4)	0.32	16 (4.2)	18 (2.9)	0.35
Secondary VF	9 (1.6)	10 (1.1)	0.55	5 (1.3)	4 (0.6)	0.45
New onset atrial fibrillation	49 (8.9)	54 (6.1)	0.05	17 (4.4)	24 (3.8)	0.76
High degree Atrioventricular Block	32 (5.8)	27 (3.0)	0.01	12 (3.1)	11 (1.8)	0.23
Asystole	17 (3.1)	28 (3.1)	1.00	8 (2.1)	6 (1.0)	0.22
CVA	8 (1.5)	4 (0.4)	0.08	0 (0.0)	2 (0.3)	0.70
Acute renal failure	40 (7.3)	58 (6.5)	0.64	13 (3.4)	17 (2.7)	0.66
Bleeding	18 (3.3)	17 (1.9)	0.14	4 (1.0)	11 (1.8)	0.52
Blood transfusions	14 (50.0)	12 (1.3)	<0.001	2 (20.0)	10 (1.6)	0.002

*Laboratory tests*						
Peak CK (U/L) Value (median [IQR])	1181.00 (569.00, 1881.00)	1281.00 (586.25, 2850.50)	0.008	566.00 (233.00,1352.00)	606.00 (257.00, 1468.00)	0.36
Troponin I Elevated	232 (88.9)	362 (84.8)	0.15	171 (88.1)	271 (82.1)	0.08
Troponin T Elevated	222 (94.1)	493 (90.1)	0.09	149 (88.7)	317 (84.8)	0.27

CHF = congestive heart failure, MI = myocardial infarction, VSD = ventricular septal defect, MR = mitral regurgitation, VT = ventricular tachycardia, VF = ventricular fibrillation, CVA = cerebrovascular accident, CK = creatine kinase, IQR = interquartile range.

**Table 4:** Treatment at discharge and at 30 day follow up over time periods of those with TIMI 0 vs TIMI 1-3.

	TIMI 0			TIMI 1-3		
	2008–2010	2013–2018	p value	2008–2010	2013–2018	p value
n	550	892		384	627	

*Treatment at discharge*						
Aspirin	519 (96.5)	840 (97.3)	0.44	369 (97.4)	606 (98.5)	0.28
P2Y12 Inhibitor	507 (94.2)	600 (95.8)	0.25	360 (95.0)	386 (95.8)	0.71
Type of P2Y12 Inhibitor during hospitalization or at discharge (%):			<0.001			<0.001
Prasugrel	3 (0.6)	527 (59.8)		0 (0.0)	335 (54.2)	
Ticagrelor	0 (0.0)	233 (26.4)		0 (0.0)	194 (31.4)	
Clopidogrel	537 (99.4)	122 (13.8)		372 (100.0)	89 (14.4)	
Statin	507 (94.1)	822 (97.0)	0.01	359 (94.5)	591 (97.5)	0.02
ACE-I/ARB	444 (82.4)	717 (86.8)	0.03	311 (81.8)	515 (89.3)	0.002
Beta blocker	442 (82.2)	708 (85.3)	0.14	319 (84.2)	494 (85.5)	0.64
Referral to cardiac rehabilitation	319 (61.9)	491 (68.8)	0.01	225 (60.8)	343 (67.8)	0.03

ACE-I/ARB: Angiotensin-converting-enzyme inhibitor/Angitensin II receptor blocker.

**Fig. 2:**
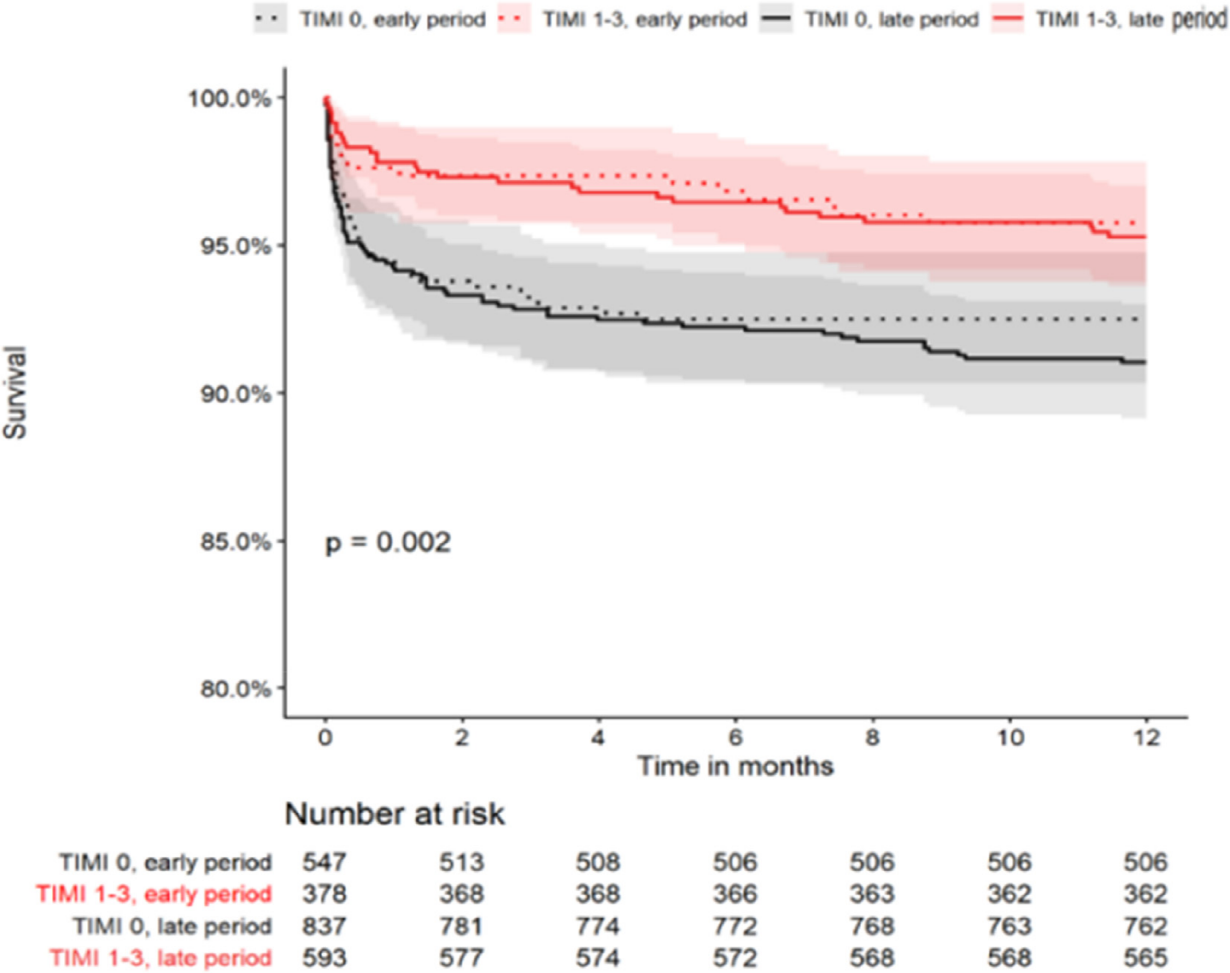
Kaplan Meier curves for 1-year mortality in patients with TIMI 0 compared to patients with TIMI 1-3 during the early period (2008–2010) and late period (2013–2018).

Overall patients presenting with TIMI 0 had worse clinical outcomes compared with those presenting with TIMI 1-3. This was evident both for 30-d MACE (12.3% vs 6.9%, P < 0.001), as well as for 1 year mortality (8.4% vs 4.5%, P < 0.001) (Table S5). Fig. 2 shows the Kaplan Meier curve for time to mortality demonstrating significantly poorer survival for those with TIMI 0 compared to those presenting with TIMI 1-3, both in the early and late period. Over time, those with TIMI 0 did not have a significant decrease in 30-d MACE or 1 year mortality. However, the rate of recurrent MI significantly decreased in the late period (2.7% vs 0.7%, P = 0.005) as seen in Table 5. The 1-year mortality of both those presenting with TIMI 0 and TIMI 1-3 did not change between periods (Table 5).

**Table 5:** Clinical Outcomes over time in those with TIMI O vs TIMI 1-3.

	TIMI 0			TIMI 1-3		
	2008–2010	2013–2018	p value	2008–2010	2013–2018	p value
n	550	892		384	627	

*30-Day clinical outcomes*						
Repeat hospitalization	91 (18.5)	117 (16.0)	0.28	59 (16.3)	86 (16.6)	0.98
Repeat MI	15 (2.7)	6 (0.7)	0.005	6 (1.6)	5 (0.8)	0.46
MACE	72 (13.1)	104 (11.8)	0.52	29 (7.6)	41 (6.6)	0.64

*Death rates*						
1-year mortality	41 (7.5)	75 (9.0)	0.38	16 (4.2)	28 (4.7)	0.84

MI = Myocardial Infarction, MACE = major adverse clinical events including death, myocardial infarction, stroke, unstable angina, stent thrombosis, urgent revascularization.

## 5 Discussion

This study based on a national ACS registry, demonstrates important insights regarding the prognostic value of the preprocedural TIMI flow grade in a real-world cohort of patients presenting with STEMI. Firstly, the majority of patients presenting with STEMI have TIMI flow grade 0. The proportion of patients with TIMI flow 0 has remained unchanged while the proportion of those with TIMI flow 3 has increased in the later period. Secondly, despite advances in PCI and pharmacotherapy, those presenting with TIMI flow 0 still have poorer early clinical outcomes compared to those with TIMI flow 1-3. However, the rate of in-hospital complications of patients with TIMI flow 0 has decreased over time, while it remained unchanged in patients with TIMI 1-3. Thirdly, the 30d MACE and 1-year mortality have not significantly changed over time in either subgroup of TIMI flow grade 0 or 1-3.

The TIMI Coronary Grade Flow was initially established to ensure a uniform method of documenting epicardial perfusion on coronary arteriography. It has proven to be an effective clinical tool. Preprocedural TIMI grade flow has been shown to stratify patients at risk for increased MACE and mortality post STEMI [3], [4], [7]. Although the clinical outcomes of STEMI patients have improved throughout the years, the prognosis of these patients has not changed equally. Our first major finding was that the proportion of patients with TIMI flow grade 0 has remained unchanged while those with TIMI flow grade 3 has increased in the later period. The diagnosis of STEMI is the clinical representation of an acute occlusion of an epicardial coronary artery and thus it is not surprising that the majority of patients with STEMI have a TIMI flow grade of 0 at the time of catheterization. What is encouraging, is that the number of STEMI patients with TIMI flow grade 3 has increased over time. This suggests that while the electrocardiographic initial diagnosis is of acute coronary artery occlusion, there is a beneficial effect of the treatment given before the time of catheterization and as such the coronary flow seen at catheterization is improved. This finding is in contrast to the findings of the ATLANTIC trial, which showed that prehospital administration of ticagrelor in patients with STEMI did not improve pre-PCI coronary reperfusion. However, in the ATLANTIC trial cohort, the median time from symptoms onset to STEMI diagnosis and from randomization to angiography were 73 and 48 min respectively. These time delays reported are significantly shorter than in our real-world cohort and thus the effect of pre-hospital ticagrelor could have been blunted in this study [8]. The increasing availability and use of ECG-mobile devices may also be help tool in the early identification and thus improved outcomes of STEMI patients [9]. Further studies are needed to validate this.

We found that patients presenting with TIMI flow grade 0 still have poorer early clinical outcomes compared to those with TIMI flow grade 1-3 and this was still valid in the late period. This is consistent with findings of previous studies showing the poor prognosis associated with a lower TIMI flow grade [2], [4]. Importantly, we found that the in-hospital complications of those with TIMI flow grade 0 has decreased over time. This is an encouraging finding which possibly suggests that the in-hospital management of these patients has improved. This could be due to improved primary PCI techniques, the use of newer-age stents and an increasing using of radial access, as well as advances in evidence-based medical management and heart failure therapies available in later years [10], [11]. Similar encouraging findings have been reported in other cohorts [12], [13]. The 30-day MACE and 1-year mortality have not significantly changed over time with either subgroup of TIMI flow grade 0 or TIMI flow grade 1-3. This observation is probably multifactorial. Firstly, our findings did not show that the time from symptom onset to primary PCI did decrease over time. This delay is a surrogate for total ischemic time and is a major determinant of outcomes [14], [15]. This delay is concerning and is due to both patient and system factors. This highlights the importance of public awareness of recognizing symptoms suggestion of myocardial infarction and accessing medical attention, as well as improving and encouraging collaboration between emergency services and cardiology services. Improvement in these aspects can assist in shortening this critical delay and thereby improving outcomes. Another explanation could be that of a survivor cohort effect. Patients who may have previously died prior to hospital transfer are benefitting and surviving due to improved advanced care in later years. However, these patients still have an increased mortality which could blunt the overall improved outcomes we would have expected in the later years. The lack of improved hard outcomes in our cohort could also be due to the size of our cohort as our results may be underpowered to detect meaningful differences in mortality.

To our knowledge this is the first study to assess temporal trends in TIMI flow grade and its effect on hard outcomes. We were able to assess this angiographic parameter and its prognostic impact in a real-world cohort, in an era in which dramatic changes have occurred in the management of patients with STEMI. Despite important advances in the management of these patients, our findings show that TIMI flow remains an important prognostic factor.

## 6 Study limitations

Our study has several limitations. Firstly, we did not have all the data on preprocedural TIMI flow grade for all STEMI patients in the ACSIS registry. Secondly, this study is an observational retrospective study with its inherent weaknesses. Thirdly, data regarding cardiac versus non-cardiac causes for mortality are not available, as well as data on the specific cause of death. Lastly, data on TIMI flow post the PCI are lacking, thus were not examined in the current study.

## 7 Conclusions

Preprocedural TIMI flow grade is still of prognostic significance in the current era. Patients with TIMI 0 have a poorer prognosis than their counterparts with TIMI 1-3. While in-hospital complications have decreased among patients with TIMI 0 over time, their 30-d MACE and 1-year mortality remained unchanged.

All authors take responsibility for all aspects of the reliability and freedom from bias of the data presented and their discussed interpretations.

## Declaration of Competing Interest

The authors declare that they have no known competing financial interests or personal relationships that could have appeared to influence the work reported in this paper.
