# Supplementary material for: Temporal trends in the pre-procedural TIMI flow grade among patients with ST- segment elevation myocardial infarction – From the ACSIS registry
Source: Int J Cardiol Heart Vasc. 2021 Sep 1;36:100868. doi: 10.1016/j.ijcha.2021.100868 (PMC8413889; doi:10.1016/j.ijcha.2021.100868)
Supplement: Supplementary Table S1 [file mmc1.docx]

Table S1: Baseline Characteristics of those with TIMI 0 vs TIMI 1-3

|  | **TIMI 0** | **TIMI 1-3** | **p value** |
| --- | --- | --- | --- |
| n | 1442 | 1011 |  |
| **Baseline characteristics** | | | |
| Age, years (median [IQR[) | 60.00 [52.00, 69.00] | 60.00 [52.00, 70.00] | 0.75 |
| Gender (male) | 1185 (82.2) | 845 (83.6) | 0.39 |
| Dyslipidemia | 969 (67.7) | 651 (64.8) | 0.15 |
| Hypertension | 776 (54.0) | 515 (51.3) | 0.19 |
| Current smokers | 731 (50.8) | 492 (48.9) | 0.36 |
| Diabetes mellitus | 439 (30.5) | 305 (30.3) | 0.95 |
| Family history of CAD | 428 (32.6) | 311 (34.1) | 0.50 |
| BMI (kg/m2), (median [IQR[) | 27.15 [24.62, 30.08] | 26.77 [24.40, 29.41] | 0.12 |
| Prior MI | 345 (24.0) | 213 (21.1) | 0.10 |
| Prior CABG | 39 ( 2.7) | 28 ( 2.8) | 1.00 |
| Prior PCI | 347 (24.1) | 220 (21.8) | 0.19 |
| Chronic renal failure | 72 ( 5.0) | 64 ( 6.3) | 0.18 |
| PVD | 68 ( 4.7) | 50 ( 5.0) | 0.85 |
| Prior CVA/TIA | 85 ( 5.9) | 75 ( 7.4) | 0.14 |
| History of CHF | 55 ( 3.8) | 24 ( 2.4) | 0.06 |
| **Prior medications** | | | |
| Aspirin | 499 (36.1) | 334 (34.6) | 0.48 |
| Clopidogrel | 95 ( 7.0) | 47 ( 5.1) | 0.07 |
| ACE-I | 319 (23.9) | 203 (22.1) | 0.33 |
| ARB | 136 (10.7) | 66 ( 7.6) | 0.02 |
| Beta blockers | 347 (26.0) | 202 (21.8) | 0.02 |
| Statins | 533 (42.8) | 351 (41.1) | 0.44 |
| CCB | 223 (17.5) | 121 (14.1) | 0.03 |
| Nitrates | 41 ( 3.3) | 14 ( 1.6) | 0.03 |
| Hypoglycemic agents | 256 (17.8) | 180 (17.8) | 1.00 |
| Diuretics | 130 (10.2) | 71 ( 8.2) | 0.15 |

CAD = coronary artery disease, IQR= interquartile range, BMI = body mass index, MI = myocardial infarction, CABG = coronary artery bypass graft surgery, PCI = percutaneous intervention, PVD = peripheral vascular disease, CVA = cerebral vascular accident, TIA = transient ischemic attack, CHF = congestive heart failure, ACE-I = Angiotensin-converting-enzyme inhibitor, ARB = Angiotensin II receptor blocker, CCB = calcium channel blockers
