# Supplementary material for: Temporal trends in the pre-procedural TIMI flow grade among patients with ST- segment elevation myocardial infarction – From the ACSIS registry
Source: Int J Cardiol Heart Vasc. 2021 Sep 1;36:100868. doi: 10.1016/j.ijcha.2021.100868 (PMC8413889; doi:10.1016/j.ijcha.2021.100868)
Supplement: Supplementary Table S2 [file mmc2.docx]

Table S2: Vital Signs on first medical contact of those with TIMI 0 vs TIMI 1-3

|  | **TIMI 0** | **TIMI 1-3** | **p value** |
| --- | --- | --- | --- |
| n | 1442 | 1011 |  |
| Killip class I | 1232 (87.6) | 893 (90.1) | 0.05 |
| Killip class II | 90 ( 6.4) | 42 ( 4.2) |  |
| Killip class III | 33 ( 2.3) | 29 ( 2.9) |  |
| Killip class IV | 52 ( 3.7) | 27 ( 2.7) |  |
| Heart rate (bpm) (median [IQR]) | 77.00 [65.00, 89.00] | 78.00 [66.00, 90.00] | 0.09 |
| Systolic Blood Pressure (mmHg) (median [IQR]) | 138.00 [119.00, 156.00] | 138.00 [120.00, 158.50] | 0.28 |
| Diastolic Blood Pressure (mmHg) (median [IQR]) | 80.00 [70.00, 92.00] | 80.00 [70.00, 93.00] | 0.50 |
| Sinus Rhythm | 1251 (86.8) | 905 (89.5) | 0.04 |
| AF/SVT | 68 ( 4.7) | 39 ( 3.9) | 0.35 |
| VT/VF | 25 ( 1.7) | 18 ( 1.8) | 1.00 |
| 2^nd^/3^rd^ degree Atrioventricular Block | 31 ( 2.6) | 12 ( 1.4) | 0.11 |
| Time from symptoms onset to primary PCI (in STEMI patients) (median [IQR]) | 184.00 [125.00, 315.00] | 194.50 [129.00, 330.00] | 0.12 |

AF/SVT = Atrial fibrillation/Supraventricular Tachycardia, VT/VF = Ventricular Tachycardia/Ventricular Fibrillation, PCI = Percutaneous Coronary Intervention, STEMI = ST-elevation myocardial infarction
