# Supplementary material for: Temporal trends in the pre-procedural TIMI flow grade among patients with ST- segment elevation myocardial infarction – From the ACSIS registry
Source: Int J Cardiol Heart Vasc. 2021 Sep 1;36:100868. doi: 10.1016/j.ijcha.2021.100868 (PMC8413889; doi:10.1016/j.ijcha.2021.100868)
Supplement: Supplementary Table S3 [file mmc3.docx]

Table S3: Angiography, In hospital complications and laboratory tests of those with TIMI 0 vs TIMI 1-3

|  | **TIMI 0** | **TIMI 1-3** | **p value** |
| --- | --- | --- | --- |
| n | 1442 | 1011 |  |
| **Angiography** | | | |
| Non-obstructive | 57 (4.0) | 47 (4.8) | 0.62 |
| 1 Vessel Diseased | 589 (41.6) | 391 (39.6) |  |
| 2 Vessel Diseased | 452 (31.9) | 314 (31.8) |  |
| 3 Vessels Diseased | 319 (22.5) | 236 (23.9) |  |
| Left Anterior Descending Artery | 667 (46.4) | 447 (47.6) | 0.64 |
| Circumflex Artery | 118 (13.1) | 145 (14.5) |  |
| Right Coronary Artery | 551 (38.4) | 357 (35.6) |  |
| Left Main | 8 (0.6) | 9 (0.9) |  |
| Saphenous Vein Graft | 16 (1.1) | 9 (0.9) |  |
| Ramus | 4 (0.3) | 5 (0.5) |  |
| Other graft | 2 (0.1) | 1 (0.1) |  |
| Bare Metal Stent | 573 (43.1) | 418 (44.5) | 0.56 |
| Drug Eluting Stent | 737 (55.5) | 534 (56.8) | 0.56 |
| **In-hospital complications** | | | |
| Composite of In-hospital complications | 433 (30.3) | 216 (21.5) | <0.001 |
| CHF mild-moderate (Killip-2) | 113 ( 7.9) | 55 ( 5.4) | 0.02 |
| Pulmonary edema (Killip-3) | 64 ( 4.4) | 37 ( 3.7) | 0.39 |
| Cardiogenic shock (Killip-4) | 91 ( 6.3) | 37 ( 3.7) | 0.005 |
| Hemodynamically significant Right Ventricular Infarction | 40 ( 2.8) | 7 ( 0.7) | <0.001 |
| Repeat MI | 13 ( 0.9) | 4 ( 0.4) | 0.21 |
| Stent thrombosis (definite/probable/possible) | 27 ( 1.9) | 12 ( 1.2) | 0.24 |
| VSD | 3 ( 0.2) | 1 ( 0.1) | 0.87 |
| MR moderate - severe | 24 ( 1.7) | 9 ( 0.9) | 0.14 |
| Pericarditis | 16 ( 1.1) | 4 ( 0.4) | 0.08 |
| Sustained VT (>125 bpm) | 38 ( 2.6) | 6 ( 0.6) | <0.001 |
| Primary VF | 55 ( 3.8) | 34 ( 3.4) | 0.63 |
| Secondary VF | 19 ( 1.3) | 9 ( 0.9) | 0.43 |
| New onset atrial fibrillation | 103 ( 7.1) | 41 ( 4.1) | 0.002 |
| High degree Atrioventricular Block | 59 ( 4.1) | 23 ( 2.3) | 0.01 |
| Asystole | 45 ( 3.1) | 14 ( 1.4) | 0.009 |
| CVA | 12 ( 0.8) | 2 ( 0.2) | 0.07 |
| Acute renal failure | 98 ( 6.8) | 30 ( 3.0) | <0.001 |
| Bleeding | 35 ( 2.4) | 15 ( 1.5) | 0.13 |
| Blood transfusions | 26 ( 2.8) | 12 ( 1.9) | 0.30 |
| **Laboratory tests** | | | |
| Peak CK (U/L) Value (median [IQR]) | 1232.00 [578.00, 2450.00] | 597.00 [250.25, 1393.75] | <0.001 |
| Troponin I Elevated | 594 (86.3) | 442 (84.4) | 0.37 |
| Troponin T Elevated | 715 (91.3) | 466 (86.0) | 0.003 |

CHF = congestive heart failure, MI = myocardial infarction, VSD = ventricular septal defect, MR = mitral regurgitation, VT = ventricular tachycardia, VF = ventricular fibrillation, CVA = cerebrovascular accident, CK = creatine kinase, IQR = interquartile range
