# Supplementary material for: Temporal trends in the pre-procedural TIMI flow grade among patients with ST- segment elevation myocardial infarction – From the ACSIS registry
Source: Int J Cardiol Heart Vasc. 2021 Sep 1;36:100868. doi: 10.1016/j.ijcha.2021.100868 (PMC8413889; doi:10.1016/j.ijcha.2021.100868)
Supplement: Supplementary Table S4 [file mmc4.docx]

Table S4: Treatment at discharge and at 30 day follow up those with TIMI 0 vs TIMI 1-3

|  | **TIMI 0** | **TIMI 1-3** | **p value** |
| --- | --- | --- | --- |
| n | 1442 | 1011 |  |
| **Treatment at discharge** | | | |
| Aspirin | 1359 (97.0) | 975 (98.1) | 0.12 |
| P2Y12 Inhibitor | 1107 (95.1) | 746 (95.4) | 0.85 |
| Type of P2Y12 Inhibitor during hospitalization or at discharge (%) |  |  | 0.07 |
| Prasugrel | 530 (37.3) | 335 (33.8) |  |
| Ticagrelor | 233 (16.4) | 194 (19.6) |  |
| Clopidogrel | 659 (46.3) | 461 (46.6) |  |
| Statin | 1329 (95.9) | 950 (96.3) | 0.64 |
| ACE-I/ARB | 1161 (85.1) | 826 (86.3) | 0.43 |
| Beta blocker | 1150 (84.1) | 813 (85.0) | 0.60 |
| Referral to cardiac rehabilitation | 810 (65.9) | 568 (64.8) | 0.64 |

ACE-I/ARB: Angiotensin-converting-enzyme inhibitor/Angiotensin II receptor blocker
