# Supplementary material for: Temporal trends in the pre-procedural TIMI flow grade among patients with ST- segment elevation myocardial infarction – From the ACSIS registry
Source: Int J Cardiol Heart Vasc. 2021 Sep 1;36:100868. doi: 10.1016/j.ijcha.2021.100868 (PMC8413889; doi:10.1016/j.ijcha.2021.100868)
Supplement: Supplementary Table S5 [file mmc5.docx]

Table S5: Clinical Outcomes in those with TIMI O vs TIMI 1-3

|  | **TIMI 0** | **TIMI 1-3** | p value |
| --- | --- | --- | --- |
| n | 1442 | 1011 |  |
| **30-Day clinical outcomes** | | | |
| Repeat hospitalization | 208 (17.0) | 145 (16.5) | 0.77 |
| Repeat MI | 21 ( 1.5) | 11 ( 1.1) | 0.54 |
| MACE | 176 (12.3) | 70 ( 6.9) | <0.001 |
| **Death rates** | | | |
| 1-year mortality | 116 ( 8.4) | 44 ( 4.5) | <0.001 |

MI = Myocardial Infarction, MACE = major adverse clinical events including death, myocardial infarction, stroke, unstable angina, stent thrombosis, urgent revascularization
